# Evidence of predation events by marine mammals at offshore wind farms

**DOI:** 10.1038/s41598-026-65167-3

**Published:** 2026-08-06

**Authors:** Anthony W. J. Bicknell, Robert Main, Samuel Gierhart, Paul Thompson, Gordon Hastie, Philippa F. C. Wright, Matthew J. Witt

**Affiliations:** 1 Hatherly Laboratories, University of Exeter, Prince of Wales Road, Exeter, EX4 4PS UK; 2 Marine Directorate, Scottish Government, 375 Victoria Road, Aberdeen, AB11 9DB UK; 3 Lighthouse Field Station, School of Biological Sciences, University of Aberdeen, Cromarty, IV11 8YL UK; 4 Sea Mammal Research Unit, School of Biology, University of St Andrews, St Andrews, KY16 8YG Scotland, UK

**Keywords:** Acoustic tracking, Gadoid fish, Marine mammals, Offshore wind farms, Predation, Prey selection, Ecology, Ecology, Ocean sciences

## Abstract

Anthropogenic structures in the marine environment alter the availability and distribution of food for marine animals with potential consequences for individual fitness, population and community dynamics. Knowledge of how marine mammals’ behaviour or foraging activities are influence by existing wind farms is essential to predict the effect of future large-scale renewable energy development on local or regional populations. During acoustic tracking of demersal gadoid fish at two offshore wind farms in Scotland, six Atlantic cod were predated by a marine mammal close to or in the vicinity of turbine foundations. The distinct change in the temperature, depth and movement pattern of the acoustic tags allowed estimations of the time and location of predation (and likely predator) and provided details of post consumption behaviour. The detection of tags (predator) at multiple turbines within a relatively short period, post consumption, suggests the targeted use of turbine foundations as foraging sites where prey fish are known to aggregate. Moreover, the bias in predation of cod over haddock, and evidence for the cod being of higher energetic quality, provide rare evidence for prey selection by a marine mammal predator(s). These data provide further evidence for how wind farms and other introduced structures influence the behaviour of marine mammals with potential consequences for individuals and populations through changes in the distribution of prey. The ultimate consequences are to be determined but need consideration with the continued development of offshore wind farm sites.

**Supplementary Information:**

The online version contains supplementary material available at https://doi.org/10.1038/s41598-026-65167-3.

## Introduction

Coastal and offshore seascapes are changing rapidly around the world with the introduction of artificial structures to harness wind, wave, and tidal energy^1,2^. Wind turbine foundations and subsea infrastructure, e.g. scour protection and cables, are known to effect the presence and distribution of marine species^3^ but how these changes will ultimately alter population dynamics, community structure, behaviour and predator/prey interactions at a local or regional scales is still to be determined.

Knowledge of how marine mammals may be affected by offshore wind infrastructure is important across European waters given they are protected under the Habitats Directive (92/43/EEC), and their consideration is a requirement for Environmental Impact Assessments (EIA) during consenting. Currently, most attention has focussed on the potential impacts of underwater noise and vessel traffic during wind farm construction and operation^4–7^. However, it is also important to understand how wind farm infrastructure may influence foraging behaviour through changes in prey availability. The distribution of fish and other prey species can be altered through aggregation effects at wind turbine foundations or subsea cables^8–13^. These in turn may have consequences for food availability, individual energy budgets and potential population effects for marine mammals^14^. Movement and inferred foraging behaviour of two species of seal (Harbour *Phoca vitulina* and grey *Halichoerus grypus)* has been shown to concentrate around wind turbine foundations and cables^15^ providing evidence for such behavioural changes. Similarly, higher densities of harbour porpoise have been recorded during operational phases at some North Sea wind farms^16^. However, direct evidence of predation around structures is lacking, and it remains possible that increased use of wind farms may also be driven by reductions in disturbance from other factors such as changes in fisheries or vessel traffic intensity^4,5^.

Acoustic telemetry is widely used to study the ecology and movements of marine animals^17^ and provides opportunities to explore how fish movement and behaviour can be altered by the introduction of offshore wind farm structures^18^. Tags are attached or inserted into individual fish and subsequently emit coded acoustic signals for several months or years. These signals can be detected by fixed hydrophone receivers at strategic locations, or on mobile receivers deployed on vessels or animals^18,19^. Tag variants also provide sensor data including depth and temperature at the time of detection, which can reveal movements throughout the water column and alterations in the ambient temperature the fish is experiencing. When assessed in combination, the temperature, depth and location data can identify changes in expected fish behaviour that could infer mortality^20,21^ or consumption by a warm-blooded predator, e.g. cetacean or pinniped^18,21,22^. Temperature of (non-acoustic) archival satellite tags has also been used to identify predation of European eels *Anguilla anguilla* by endothermic fish (10 °C increase from ambient) or marine mammals (> 30 °C) during annual migration^23,24^.

Here, data from a study of the movements of two demersal (‘live and feed near the seabed’) gadoid fish species around northern North Sea offshore wind farms were used to explore patterns of marine mammal predation. The study area is known to support a range of marine mammal species, with harbour seal, grey seal and harbour porpoise *Phocoena phocoena* occurring most commonly across this region^25–27^. The primary aim was to use fish acoustic tag detection and sensor data to provide direct evidence of marine mammal foraging activity. In addition, these data were explored to assess whether they could provide insights into the identity of the predators and/or evidence of prey selection.

## Results

### General detection patterns of tagged fish

Of the 217 tagged fish, 104 were detected for 14 days or more (either contiguous or non-contiguous) and used in subsequent analysis. Of the 104 fish, 57 (~ 55%) were tagged in Beatrice and 47 (~ 45%) in Moray East wind farm. These included 7 cod and 28 haddock captured in 2022, and 8 cod and 61 haddock captured in 2024. The overall mean length for the 15 tagged cod was 30 cm (range 24–36 cm), and for the 89 haddock was 31 cm (range 24–38 cm) (Supplementary Table S2). Once filtered for potential false detections, the array receivers recorded 2,285,988 detections of which 1,142,783 contained temperature data and 1,143,205 contained depth data. The maximum detection period for fish tagged in April 2022 was 422 days, in June 2022 was 301 days, and in April 2024 was 396 days (Table 1). The detections for individual cod and haddock tagged in each year overlapped in time and the array clusters on which they were recorded (Supplementary Table S2; Supplementary Figure S3 & S4).

**Table 1:** Detection summary of 104 Atlantic cod *Gadus morhua* and haddock *Melanogrammus aeglefinus* tagged in 2022 and 2024.

Species	Tagged year	Tagged month	*n*	First detection	Last detection	Detection duration in days (mean)	Detection duration in days (range)	Days detected (mean)	Days detected (range)
Gadus morhua	2022	April	6	2022-04-21	2023-06-06	234	47–411	160	14–293
	2022	June	1	2022-06-24	2023-06-18	360	–	301	–
	2024	April	8	2024-04-25	2025-03-22	166	68–330	67	48–95
Melanogrammus aeglefinus	2022	April	24	2022-04-20	2023-06-16	266	25–422	158	23–392
	2022	June	4	2022-06-24	2023-05-02	155	73–313	94	67–122
	2024	April	61	2024-04-23	2025-05-29	242	29–396	149	14–366

### Predation events on tagged fish

**Fig. 1:**
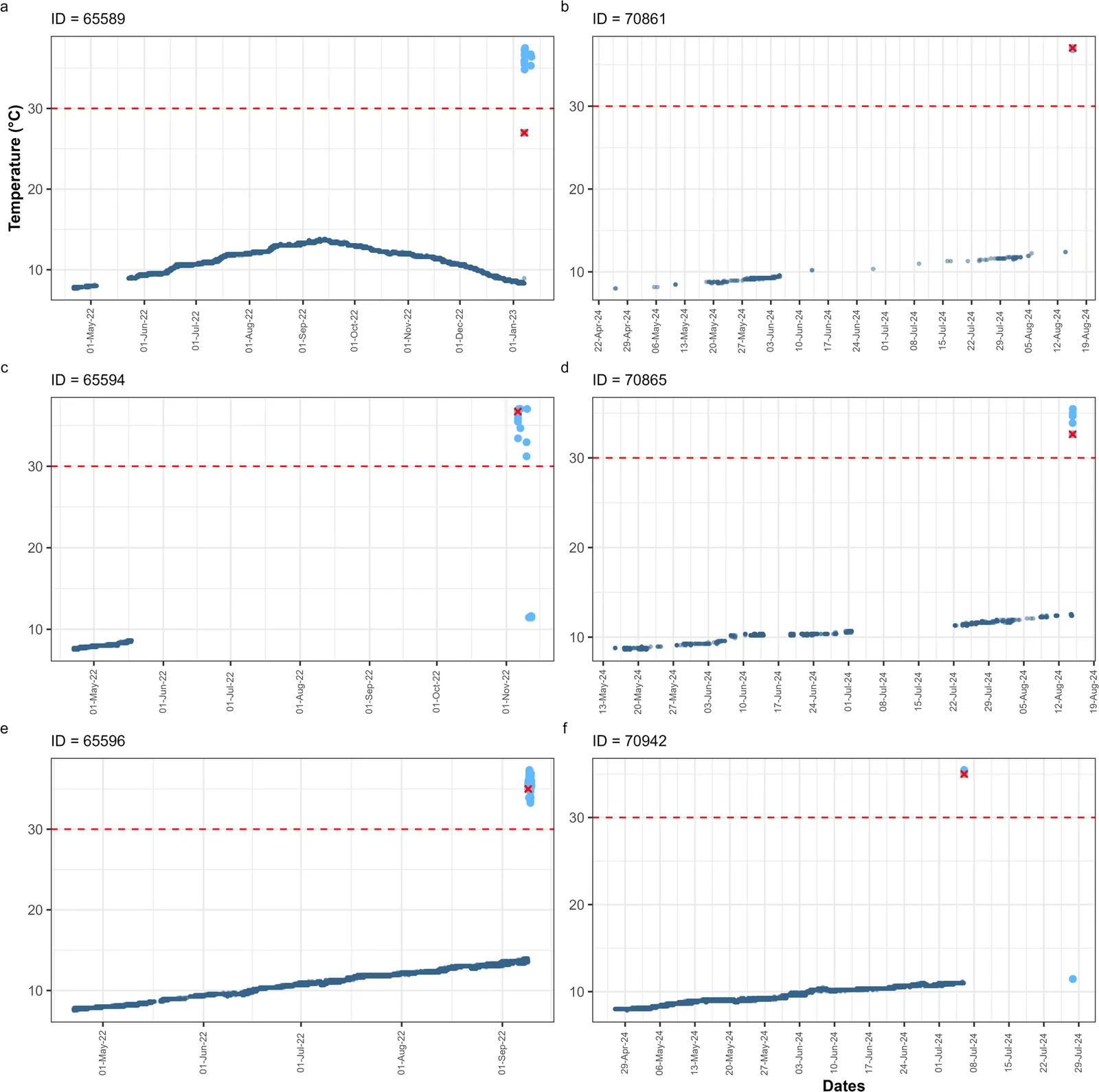
Variation in tag temperature through time for six tagged fish where an increase in tag temperature above 30 °C (shown by the red dashed line) confirmed predation by a marine mammal. Dark blue circles = fish (pre-predation), light blue circles = mammal/external (post-predation), red cross = first detection after 10 °C tag temperature increase.

Detections where the tag temperature exceeded 30 °C indicated that six of the 104 fish detected for more than 14 days were predated by a marine mammal: three in the 2022 cohort of tagged fish and three in the 2024 cohort (Fig. 1; Supplementary Figure S4). In all but one case, tagged fish were detected regularly within the wind farm up until the point at which the temperature increased to 30 °C. In the remaining case (ID 65594; Supplementary Figure S5c), the fish appeared to have left the study array for around five months before the tag, at this point registering a temperature of > 30 °C, was again detected on the array. In two cases (ID 65594 & ID 70942), tags were subsequently detected at ambient temperature after periods above 30 °C (Fig. 1c & f; Supplementary Figure S5c & S6d), indicating potential expulsion of the tag by the predator through regurgitation or defecation.

**Fig. 2:**
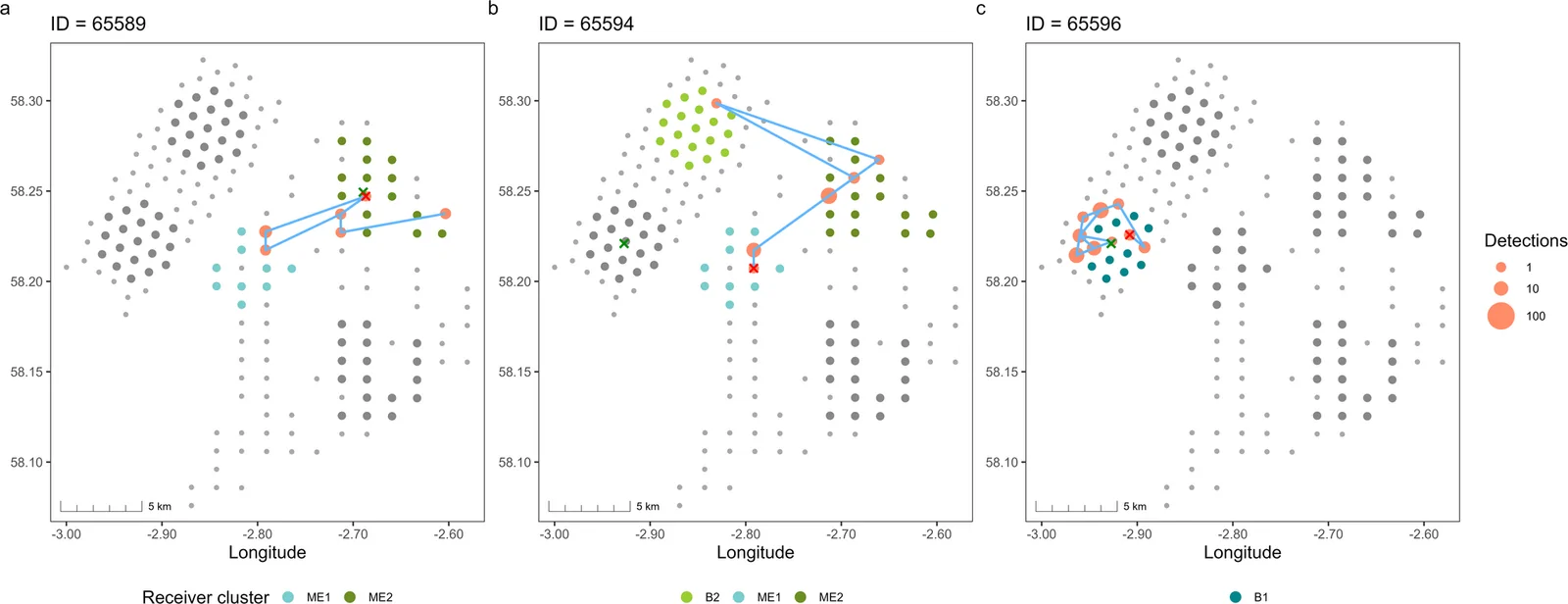
Locations of tags where repeated detections with temperature readings of above 30 °C indicated that they were within the stomach of a marine mammal. Data are shown for the three tags with at least 20 detections, over periods of (**a**) 4 days, (**b**) 6 days and (**c**) 24 h. Green cross = release location, red cross = location of first detection after 10 °C tag termperature increase, light blue line = mammal movement based on subsequent detections.

Tags registering > 30° C were recorded at multiple receivers within both offshore wind farms (Fig. 2). In four of the six cases, a rapid increase in tag temperature occurred within 3 h of a prior detection at ambient sea water temperature, either at the same receiver station (ID 65589 & 70865) or at another receiver in the same cluster (ID 65596 & 70942), providing evidence for the location and approximate time of predation (Table 2; Supplementary Figure S6 and S7). The two remaining tags had a longer delay between the temperature change detections (Table 2; Supplementary Figure S6 and S7). Tag 70861 was detected within the same array cluster after 1.7 days, indicating that predation could have recently taken place within the wind farm (Supplementary Figure S6b). In contrast, tag 65594 was detected with an elevated temperature several months after the previous detection at ambient sea water temperature, providing little insight on where or when predation occurred (Supplementary Figure S5c & Sd).

**Table 2:** Time and location of detection immediately before and when tag temperature changed by > 10 °C for six tagged Atlantic cod *Gadus morhua*. Time difference represents the time in decimal hours between the two detections.

Animal ID	Species	Before detection	Before station	Before cluster	Change detection	Change station	Change cluster	Temperature change	Time difference
65589	Gadus morhua	2023-01-07 06:24:57	ME-H18	ME2	2023-01-07 06:55:23	ME-H18	ME2	8.9 °C → 27 °C (+ 18 °C)	0.5 h
65594	Gadus morhua	2022-05-17 21:31:03	BE-E03	B1	2022-11-06 03:23:22	ME-D14	ME1	8.6 °C → 36.7 °C (+ 28.1 °C)	4133.9 h
65596	Gadus morhua	2022-09-08 21:57:01	BE-E06	B1	2022-09-08 23:05:37	BE-F05	B1	13.7 °C → 35 °C (+ 21.3 °C)	1.1 h
70861	Gadus morhua	2024-08-13 23:04:48	ME-I20	ME2	2024-08-15 15:58:56	ME-J17	ME2	12.4 °C → 37 °C (+ 24.6 °C)	40.9 h
70865	Gadus morhua	2024-08-14 15:03:11	ME-H19	ME2	2024-08-14 17:47:31	ME-H19	ME2	12.4 °C → 32.6 °C (+ 20.2 °C)	2.7 h
70942	Gadus morhua	2024-07-05 21:36:46	BE-F05	B1	2024-07-06 00:15:07	BE-G03	B1	11 °C → 35 °C (+ 24 °C)	2.6 h

### Movements of predators following ingestion of tagged fish

Following ingestion by predators, tags were detected from 4 to 123 times on one to five days (Table 3). Although limited in extent, data from three tags with > 20 detections provide evidence that predators moved between turbine locations over these relatively short time scales (Fig. 2; Supplementary Figure S7). Tag 65589 provided a ~four day detection period after temperature increase (Table 3), with detections on six receivers located between ~ 1.13–6.5 km apart across two array clusters (Fig. 2a; Supplementary Figure S7b). Tag 65594 was detected for the longest post temperature increase (~ six days; Table 3) at six receivers at distances from ~ 1.12 km within a cluster to over 10 km between the different clusters (Fig. 2b; Supplementary Figure S7d). Finally, tag 65596 was detected for 24 h after the temperature increase on nine different receivers that were located between ~ 1.16–3.1 km apart in array cluster B1 (Fig. 2c; Supplementary Figure S7f).

**Table 3:** Detection, depth and temperature details before and after putative predation for six tagged Atlantic cod *Gadus morhua*.

Animal ID	Scientific name	Release year	Predation event	First detection	Last detection	Days (dates) detected	Days (24 h) at large	Detection receivers	Receiver changes	Receiver changes per day	Detections	Depth detections	Depth (min-max)	Depth (var)	Temperature detections	Temperature (min-max)
65589	*Gadus morhua*	2022	Before	21-04-2022	07-01-2023	245	261.3	10	82	0.3	79,184	39,414	−37.9–53.3	0.7	39,770	7.7–13.8
			After	07-01-2023	11-01-2023	2	4.1	6	7	1.7	28	13	−0.6–49.3	302.4	15	27.0–37.5
65594	*Gadus morhua*	2022	Before	22-04-2022	17-05-2022	26	25.9	6	33	1.3	5,016	2,562	−19.3–45.7	5.4	2,454	7.5–8.6
			After	06–11-2022	12-11-2022	5	6.0	6	5	0.8	68	32	−0.9–53.3	444.7	36	11.5–37
65596	*Gadus morhua*	2022	Before	22-04-2022	08–09-2022	140	139.9	3	131	0.9	38,851	19,529	−39.4–46.9	1.0	19,322	7.5–14.0
			After	08–09-2022	09-09-2022	2	1.0	9	24	24	123	62	−0.6–46.0	272.2	61	33.3–37.3
70861	*Gadus morhua*	2024	Before	25-04-2024	15-08-2024	48	111.7	5	39	0.3	435	187	−5.4–51.5	11.2	248	8.0–12.4
			After	15-08-2024	15-08-2024	1	0.0	1	-	-	4	2	−50.6–51.2	0.2	2	36.9–37.0
70865	*Gadus morhua*	2024	Before	15-05-2024	14-08-2024	61	91.3	11	194	2.2	4,035	2,055	−39.7–51.2	8.7	1,980	8.6–12.6
			After	14-08-2024	14-08-2024	1	0.1	2	1	-	13	7	−0.6–37.0	241.9	6	32.6–35.5
70942	*Gadus morhua*	2024	Before	27-04-2024	06–07-2024	71	70.0	5	608	9.3	34,063	16,986	−0.3–43.9	0.9	17,077	7.8–11.1
			After	06–07-2024	27-07-2024	2	21.8	2	1	-	5	1	−0.3–0.3	-	4	11.5–35.5

Detection receivers = number of receivers detected on; Receiver changes = number of times detections changed to another receiver; Changes per day = receiver changes/days at large.

**Fig. 3:**
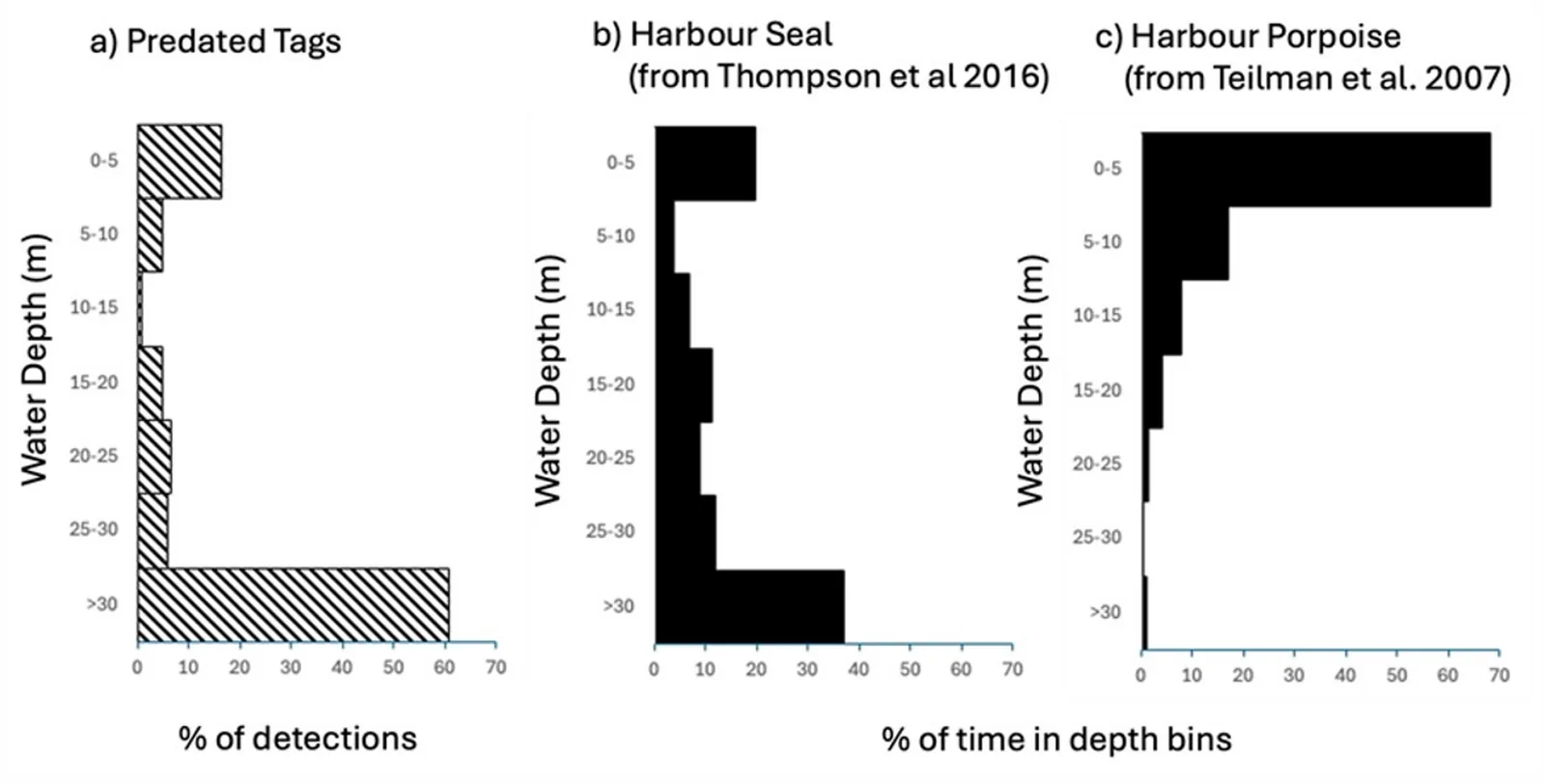
(**a**) Depth distribution of all detections of tags registering > 30 °C which were assumed to have been predated and transmitted from within a marine mammal. These can be compared with available data on the time that potential predators may be spending at different depths when foraging in similar shallow coastal environments. (**b**) Re-presents data from Fig. 7 in Thompson et al.^48^, which were collected from 14 GSM-GPS tagged harbour seals diving in the Pentland Firth, Scotland. (**c**) re-presents data from Fig. 6 of Teilmann et al.^50^, collected from 14 porpoises equipped with satellite linked time-at-depth recorders that were diving in Danish coastal waters.

Data on the depth of tags following predations were sparse, with between one and 68 depth detections per tag (Table 3). The overall depth distribution of all detections from tags registering > 30 °C shows that most tags were detected while at depths below 30 m, at or close to the seabed around the receivers (Fig. 3a).

**Fig. 4:**
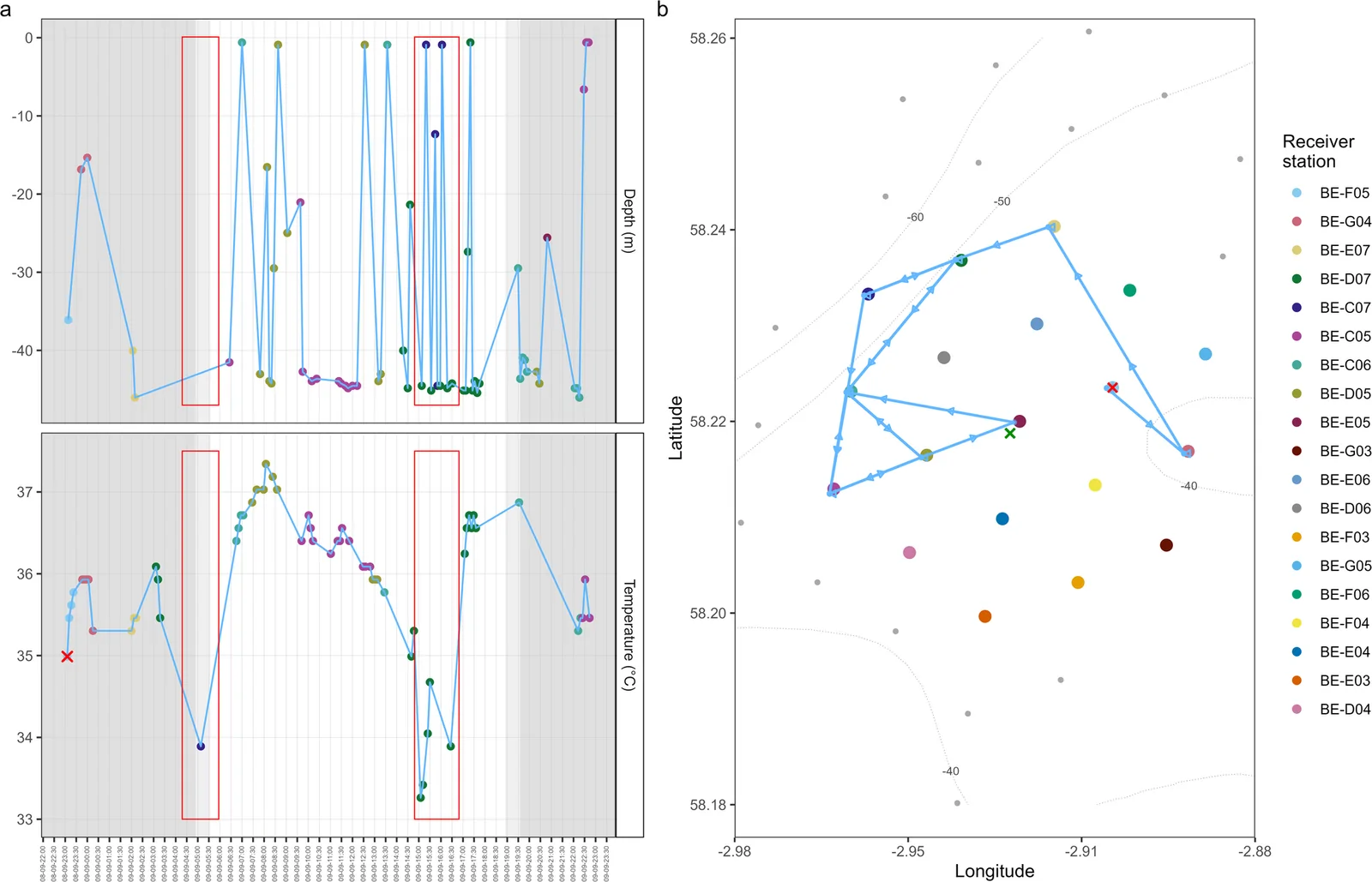
(**a**) Depth and temperature timeseries panels for tag 65596 after predation (dark grey shading = night, light grey = dawn/dusk, red rectangles = areas of notable temperature reduction and associated depth detections). (**b**) Spatial illustration of detection locations across the B1 receiver cluster for tag 65596 after predation (small light grey filled circles = wind turbines, black dot = detection location (amount not shown), light blue line with direction arrows = mammal movement). Coloured filled circles = receiver station (see legend), green cross = cod release location, red cross = first detection after consumption by marine mammal.

Only one tag (65596) provided regular depth readings through the 24 h over which sustained high temperatures indicated that it remained within the predator. These revealed eight vertical ascents to the surface with subsequent return to below 40 m (Fig. 4a), and detections on nine different receivers located between ~ 1.16–3.1 km apart in array cluster B1 (Fig. 4b). The temperature detections showed variation post increase with two noteworthy declines below 34 °C, one of which coincided with 3 vertical movements from the surface to below 40 m (Fig. 4a).

### Prey selection

All six observed predation events involved tagged cod (6 of 15 tagged cod). No haddock were identified as being predated during their detection periods (0 of 89 tagged haddock). The probability of detecting zero haddock predation events based on the observed rates was very low (Fishers exact test: p-value < 0.0001; Supplementary Table S3), suggesting a bias towards predation upon tagged cod given the high predation rate (0.4).

**Fig. 5:**
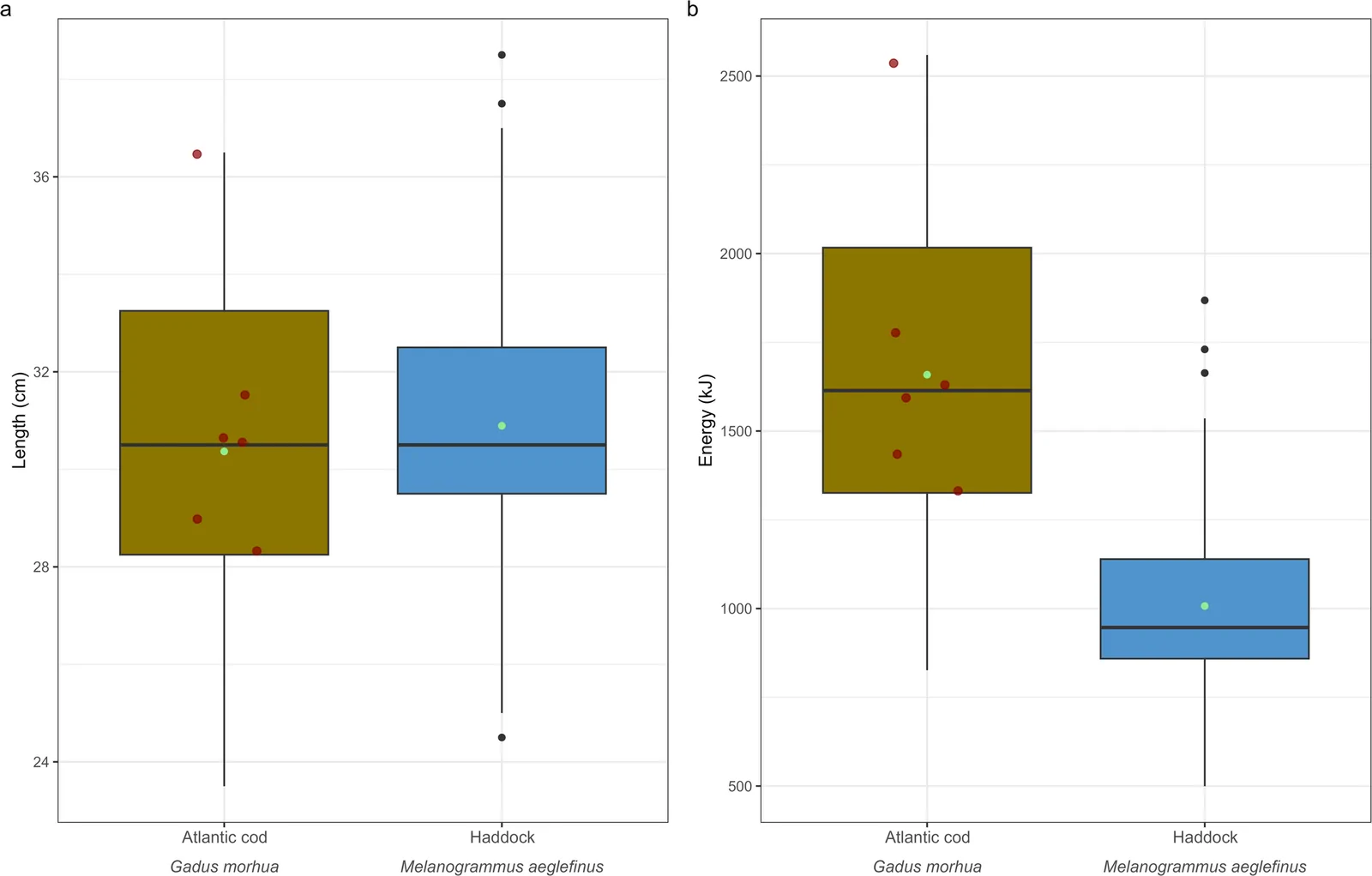
Box and whisker plots for length (**a**) and estimated energy (**b**) of all tagged Atlantic cod and haddock. Red dots = predated Atlantic cod; Green dots = mean values.

There was no significant difference between the lengths of all tagged cod or just predated cod, and haddock (tagged cod vs. haddock: p-value 0.54; predated cod vs. haddock: p-value 0.88; Fig. 5a). However, there was significant higher energy estimates for tagged cod or predated cod when compared to haddock (tagged cod vs. haddock: p-value < 0.0001; predated cod vs. haddock: p-value < 0.0001; Fig. 5b), indicating cod would be of higher quality (provide more energy) for predators.

## Discussion

Changes in temperature, depth profile and spatial movement of 104 cod and haddock acoustically tagged and released within two operational wind farms indicated that 6 cod had been predated by a marine mammal. Two of these events could be identified as taking place close to wind turbine foundations (< 400 m based on detection range) were the fish where in proximity, and location detections post-consumption of one cod revealed putative attendance at multiple turbine foundations in quick succession suggesting prospecting. To our knowledge, this is the first direct evidence of marine mammal predation of fish at wind turbine foundations, and further evidence that they represent foraging sites for some individuals^15^.

### Proximity to turbine foundation and foraging

The probability of detecting the acoustic tag transmissions on a receiver within the OWF array was high (> 85%) up to 400 m away and declined sharply between 400 and 600 m (Supplementary Figure S2). The combination of range test results and removing detections on more than one receiver within a short interval, provided confidence in that most tag detections were within 400 m of a receiver deployed next to a turbine foundation. Finer scale position data (i.e. accuracy greater than 400 m) are not available using the array design deployed in this study, and as such closer attendance of the fish or marine mammal to the turbine foundation is not certain. However, it is known that cod and haddock are attracted to within 100 m of turbine foundations due the reefing effect that creates hard substrate habitat and/or patches of enhanced prey availability in soft sediment environments^8,28–30^. Cod have been particularly well studied at turbine foundations in the southern North Sea, with evidence of strong attraction and residency within 50 m or less of the structures^28,31^. Similar residency behaviour has been found close to hard substrate artificial reefs^32^, and much higher abundance within 100 m has been observed at an oil and gas platform^33^. Three of the six predated cod (65589, 65596, 70942; Supplementary Figure **S**5 & S6) demonstrated periods (months) of consistent detections on single receivers, with only limited detections on neighbouring receivers signifying local residency and proximity to the turbine foundations. After being predated upon, the detections for two of these tags contrasted with the previous local residency behaviour, with one being detected on nine receivers within a day (Fig. 2; Table 3), indicating the predator was moving throughout the wind farm, and coming in proximity to other turbine foundations. Furthermore, two shorter periods of lower temperature for this individual (Fig. 4) may be related to prey and ambient water entering the stomach during subsequent capture events while foraging^22,34,35^, which also align with depth profiles of dive behaviour (Fig. 5).

### Marine mammal predator

The primary identifier of predation by a marine mammal was the marked increase in tag temperature from periods of temperature equivalent to the ambient water. Several marine mammal species frequent the Moray Firth region, but there are four species that are most likely to occur within these wind farm sites: harbour porpoise^26^; grey seal and harbour seal^27^; and bottlenose dolphin *Tursiops truncatus*^36^. Cod have been identified in the diet of all four of these species, both in the Moray Firth^37–40^ and other regions of the North Sea^37–44^. However, other cetaceans such as white-beaked dolphins *Lagenorhynchus albirostris*, common dolphins *Delphinus delphis*, Rissos’ dolphins *Grampus griseus* and killer whales *Orcinus orca* are occasionally recorded in the Outer Moray Firth^45,46^, and all these could potentially be predating upon this size class of cod.

Whilst the body temperature of different marine mammals may vary slightly^47^, tag temperatures cannot be used to differentiate between potential species of marine mammal predators. Similarly, neither predation events (Table 3) nor the presence of different marine mammals demonstrates repeated seasonal patterns, so this did not provide insights to the potential identity of the predators. Low tag transmission rates (~ 3/minute) also limit the potential to relate the time-series of acoustic detections to dive profiles of potential predators. Nonetheless, available data suggest that the depth distribution of predated tags is more likely to represent that of a diving seal than a small cetacean (Fig. 3). Information on the amount of time that marine mammals spend at different waters depths is available for a limited number of species. However, dive profiles of harbour seals and grey seals indicate that they generally feed close to the bottom and spend much of the dive at depth when in shallow coastal waters e.g^39^. This is illustrated in Fig. 3b using data for harbour seals taken from Thompson et al.^48^, which provides information on the proportion of a predator’s time that is spent at different water depths. In contrast, harbour porpoises and other small cetaceans typically spend much of their time in the upper layers of the water column in similar shallow coastal waters^49–51^. For example, Teilmann et al.^50^ showed that tagged harbour porpoises spent 45–63% of their time in the upper 2 m, and depth distributions of porpoises taken from their study highlight that much less time is spent at depth compared to seals (Fig. 3c). Without further evidence and sampling it is not possible to confirm the identity (or identities) of the species responsible. However, pinnipeds have been found to interact with OWF turbine foundations and cable infrastructure^15,52^, and the movement of local harbour and grey seals are known to overlap with the Moray Firth study sites^53^. The size of predated cod also align well with the suggestion that harbour seals in the Moray Firth may prefer cod of ~ 30 cm^54^, and cod had become more prevalent in their diet when analysed over a ~ 20 year period (1987–2011)^55^. Based on the depth profiles of tag detections here and from previously tagged seals and porpoises, plus the dietary evidence from local populations, we suggest that tagged fish were most likely to be predated by either harbour or grey seals. However, we cannot exclude the possibility that some fish were taken by other predators such as harbour porpoises given they are known to forage close (< 200 m) to older structures in this area^56^.

### Evidence for prey selection

The sample of tagged fish available to marine mammal predators at the study site was heavily skewed towards haddock, yet all the fish that were predated were cod. Thus, although the identity of the predators remains uncertain, these data provide rare evidence for prey selection in a wild marine mammal (discussed below).

Prey acquisition is a crucial component of marine mammal bioenergetics, balancing foraging effort (efficiency) with ingested energy required for survival and reproduction^57,58^. Targeting prey with an optimal balance between energetic quality and capture effort is therefore expected (Optimal foraging theory^59^ and the preferential selection of certain species and/or age classes by marine mammals^51,60,61^ means they are likely to have an important role in shaping marine communities^62,63^. To date, evidence for prey selection has been constrained by uncertainties over the true availability of different prey to marine mammal predators. For example, there is known to be potential bias in fisheries surveys where these are used to characterise prey availability for comparison with marine mammal diet^60^. Previously, the only studies which have precise data on the relative abundance of different prey species are those that have directly fed animals either in captivity^64^ around fisheries vessels^58^. Although previous diet studies indicate that marine mammal populations generally consume a wide range of species, individuals often show distinct preferences for particular species when offered a choice of prey^58^. Our data suggest that the marine mammal(s) foraging at this wind farm site showed a preference for cod over haddock based on the absence of haddock predation compared to a 0.4 predation rate of cod in the tagged sample. The sample of tagged fish was skewed ~ 1:6 in favour of haddock using the rod and line capture method, and when this ratio was compared to abundance data from two different sampling methods used in the Moray Firth study site it was found to be representative (potentially conservative) of the higher relative abundance of haddock compared to cod: pre-OWF construction trawl catch surveys in the Moray Firth, the species ratios were ~ 1:770 in 2019^65^ and ~ 1:3860 in 2021^66^; and observations from baited underwater camera surveys in and around the OWFs found the ratio to be ~ 1:80 in 2022^8^ and ~ 1:262 in 2024^67^. The sample size ratio does not influence the result of the predation probability test, but corroborating evidence of a larger haddock population suggests the reduced relative availability of cod as prey in the study sites. The cod and haddock tagged in this study did not differ significantly in length, but energy estimates where significantly higher for cod (predated or not) indicating prey quality could be a reason for this apparent selection. The length-weight relationship and energy estimates were derived from a relatively small number of cod from the study area, so could be viewed with caution. However, the difference holds when using additional samples (haddock = 26; cod = 10) from another North Sea site as part of same energetic study^68^ suggesting a reliable relationship. Selection could also be influenced by differences in the behaviour of the prey species that affect catchability, or other functional traits affecting choice^69^. Seasonal migration or movement away from the wind farms could create divergence in availability of the two species, but there was no strong evidence for this behaviour when these data were more broadly analysed (results not included; A.W J. Bicknell, pers. comm). This is likely a consequence of the relatively young age structure of the tagged fish and local populations (1–2 age cod; 1–4 age haddock)^8,67^. A synergistic effect of quality and behaviour of prey may enhance selection for certain species and could be evident for cod in this study, which had higher energetic estimates and show residency to a relatively small area close to a few (sometimes one) turbine foundations. Nevertheless, this new evidence for the likely existence of prey selection for cod is important to consider when assessing predator-prey dynamics and interactions between competing predators^55^ or with commercial fisheries^36,43^.

## Conclusion

The most likely predators of the tagged cod in this study are seals (grey or common) that commonly occur in the Moray Firth and have been observed and tracked in the offshore wind farm boundaries^27,70^. The predation events occurred close to wind turbine foundations where cod have been shown to often reside, attracted by the hard substrate structures and possible enhanced feeding opportunities^28^. Lack of predation of the more numerous haddock suggests prey selection, and the higher energetic estimates for the cod provide support for optimal foraging of high-quality prey. The apparent prospecting of one seal post consumption of the tagged cod, provide evidence that offshore wind farm jacket foundations offer feeding opportunities for predators by aggregating prey species. The small dataset and single location mean generalisation of this behaviour would be speculative, with further observations and study sites required to infer broader occurrence at wind farms. The consequences of this behaviour for prey and predator populations are still to be determined in the Moray Firth but, if a widespread behaviour, will need consideration for other populations with the continued large-scale development of offshore wind farms.

## Methods

### Study location

**Fig. 6:**
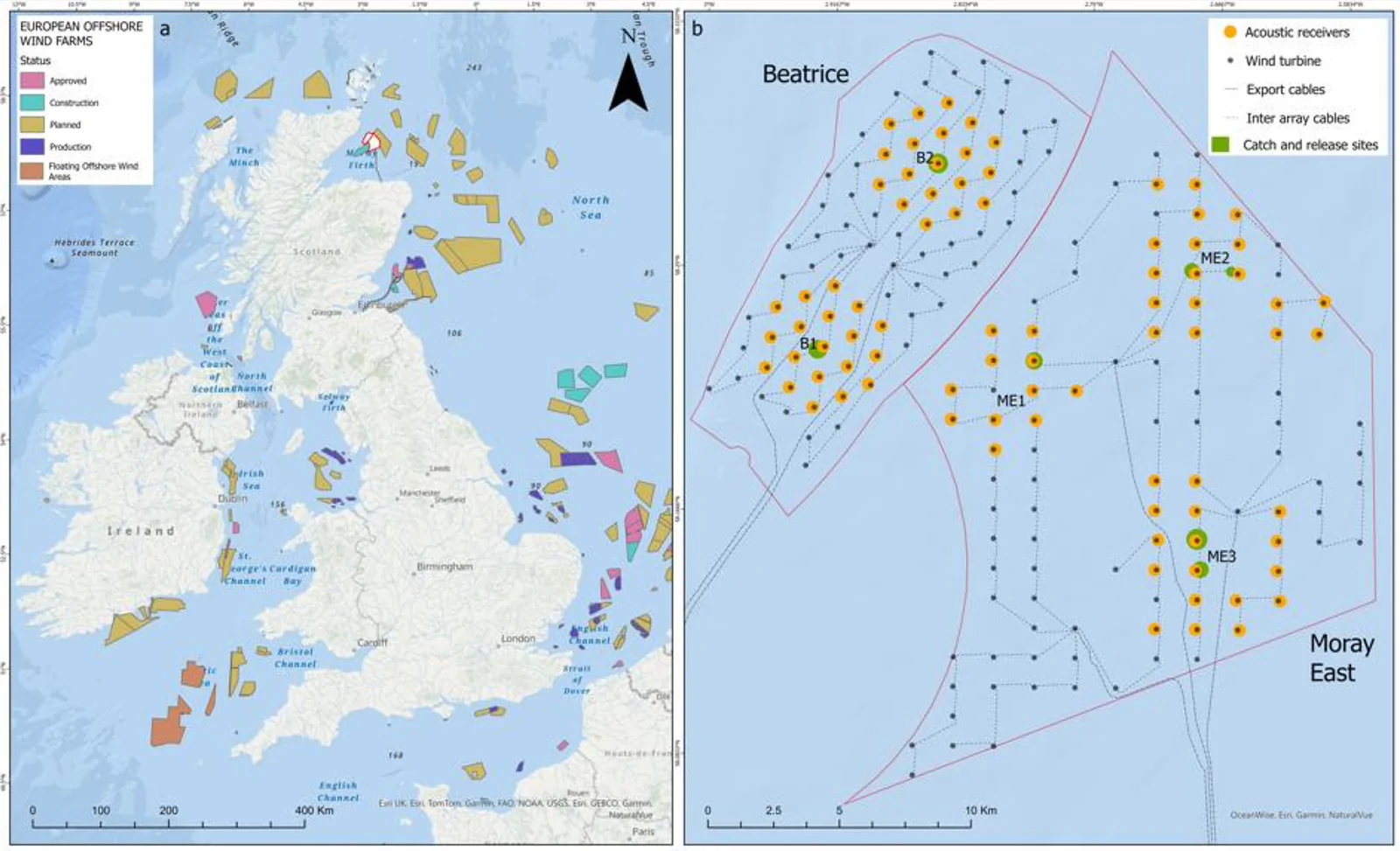
(**a**) Offshore wind farms around the UK and status as of 25th August 2025 (EMODnet Human Activities Portal, https://emodnet.ec.europa.eu/en/human-activities). Beatrice and Moray East operational wind farms indicated in white with red boundaries. (**b**) Beatrice and Moray East wind farm boundaries with turbine and cable configuration. Location of acoustic receivers (array) indicated with orange filled circles with names of five array clusters.

The study took place within the Beatrice and Moray East offshore wind farms in the Moray Firth, Scotland (Fig. 6). The wind farms are located 7.5–23 nm offshore in water depth of 35–60 m. Beatrice operates 84 7 MW turbines with four leg jacket foundations that were installed between August 2017 and July 2018. Moray East operates 100 9.5 MW turbines with three leg jackets that were installed between July and December 2020. Neither site has scour protection at the base of the jacket structures. Commercial fishing is permitted within the wind farm boundaries, with scallop dredging, trawling and creel potting known to take place^71^, sometimes close to the turbines (< 50 m). The seabed substratum across the wind farms comprised two biotopes: medium to fine sands and muddy sands; or cobbles and pebbles, gravels and coarse sands^72^.

### Acoustic telemetry array and tagging

In March 2022 an array of 84 Innovasea (http://www.innovasea.com) VR2AR receivers (69 kHz) was deployed in the Beatrice and Moray East wind farms. The receivers were positioned 50 m northeast of a wind turbine foundation in five clusters spread across the two wind farms (two in Beatrice and three in Moray East: Fig. 6b). The mean distance between receivers in the clusters was: B1 = 1.16 km, B2 = 1.16 km, ME1 = 1.55 km, ME2 = 1.53 km & ME3 = 1.52 km. Receivers were deployed with ~ 65 kg of chain ballast and moored using an acoustic release canister (RS Aqua ARC; http://www.rsaqua.co.uk). The array was maintained by replacing receivers and downloading detection data every six to 12 months (depending upon weather) between deployment in March 2022 until final retrieval in June 2025.

Fish were captured by rod and line within the Beatrice and Moray East wind farms and tagged with Innovasea V9TP 2 × (69 kHz) acoustic transmitters during 2022 (April and June) and 2024 (April). In total, 21 Atlantic cod *Gadus morhua* (cod) (2022 *n* = 7, 2024 *n* = 14) and 196 haddock *Melanogrammus aeglefinus* (haddock) (2022 *n* = 46, 2024 *n* = 150) were tagged. Prior to tagging, fish were held in aerated holding tanks before being transferred to a shallow anaesthetic bath dosed with 140 mg/1l MS222. Once anaesthetised fish were measured and placed dorsally on a V-shaped cradle, where they were ram-ventilated with maintenance anaesthetic dose of MS-222. The acoustic transmitters were inserted into the peritoneal cavity via an incision (10–15 mm) and closed using two absorbable monofilament sutures. Lidocaine analgesic (2% spray) was topically applied to the surgical site prior to incision. After the surgical procedure, fish were transferred to a recovery tank, held for a short period prior to decompression becoming apparent and returned to the seabed in a release cage that opened 2–3 h after descent.

Fish tagging activities were reviewed and signed off by University of Exeter Ethical Review Committee. All procedures were performed in accordance with UK Home office project licences held by Scottish Marine Directorate and University of Exeter (PP9073566). and personal individual licences held by project team members (PILh). These licence permits, and procedures within, are regulated under the UK Animals (Scientific procedures) Act 1986.

### Tag data, range test and analyses

The V9TP 2X tags transmit their unique identity code with associated temperature or depth (pressure) data alternately every 180 s (150 minimum – 210 maximum intervals). When within detection range, data from these detections were stored on the VR2AR receiver until download during maintenance cruises. Range detection tests conducted within the study array indicate that there was a high detection probability (≥ 80%) at distances ≤ 400 m from receivers and near-zero detection probability (≤ 1%) beyond 750 m (see Supplementary Materials for full methods; Supplementary Figure S2).

Downloaded detections from implanted tags were first filtered to remove potential false detections when either: (1) only a single detection was received in a day; (2) detections were heard on two or three different receivers within 2.5 min or (3) and detections fell outside realistic intervals between detections considering distances, elapsed time and known maximum speed for each species. In this study, only those fish that were detected within the study array on at least 14 days were used in subsequent analysis.

### Predation identification

Tag temperatures were expected to track the surrounding water when within an ectothermic fish. However, following previous work by Righton et al.^23^, if a tag detection had a temperature value that exceeded 30 °C, the fish was assumed to have been consumed by an endothermic marine mammal (~ 37 °C^47^).

For those cases where a tag temperature of > 30 °C demonstrated that a predation event had occurred, the time-series of detection locations, depths, and temperatures was further explored to investigate the nature of these likely predator prey events. First, the pattern of temperature and locations were used to determine whether predation may have occurred within the wind farms. Second, the distribution of depths before and after predation were compared with available data on dive profiles of seals and small cetaceans to explore the identity of the predator.

### Prey selection

The predation probability of tagged cod and haddock detected for over 14 days in this study (i.e. 15 cod vs. 89 haddock) was expected to be the same, given no *a priori* evidence to suggest otherwise. If marine mammal predators exhibit no preference between these prey species, then detections of predation events would be expected to occur at the same ratio. A one-tailed Fisher’s exact test was used to compare whether observed predation events differed from expected.

Factors that might influence prey selection within the sample of tagged fish were also explored. First, species differences in the length of tagged fish were compared. These data were then used to explore potential species differences in calorific content using energy values of local cod and haddock of known size. Prey energy density (kJ/g) values were obtained using standard bomb calorimetry methods of 56 (haddock *n* = 40, cod *n* = 16) fish samples collected in summer and autumn (periods of predation events) of 2021 to 2024 within the study area for related studies of prey energy landscapes. These were used to derive length–total energy (kJ) relationships^68^ that were applied to the measured lengths of the tagged fish to estimate their total energy content. Analysis of variance (ANOVA) models were used to compare length and energy between tagged cod and haddock, and predated cod and haddock.

All data processing and analysis was implemented in R version 4.4.1 using Tidyverse packages for data manipulation and visualization^73^, and splines for flexible modelling^74^.

## Supplementary Information

Below is the link to the electronic supplementary material.

Supplementary Material 1

## Data Availability

Data available via designated repository: [https://doi.org/10.6084/m9.figshare.31860610](https:/doi.org/10.6084/m9.figshare.31860610).
