# Supplementary material for: Evidence of predation events by marine mammals at offshore wind farms
Source: Sci Rep. 2026 Aug 6;16:28351. doi: 10.1038/s41598-026-65167-3 (PMC13562624; doi:10.1038/s41598-026-65167-3)
Supplement: Supplementary file 1 [file 41598_2026_65167_MOESM1_ESM.docx]

**Supplementary materials**

**Acoustic tags range test**

***Method***

To characterise the tag detectability a range test was conducted using data from four V9 detection only tags deployed ~1m above the seabed at predefined distances (100m, 200m, 300m, and 400m) from a VR9AR acoustic receiver in cluster B2 for ~1 year. Additional detection data were also from two other receivers in the B2 cluster that were within 1.15km of the four V9 tags. Precise distances between each tag and receiver latitude and longitude coordinates were calculated using the distHaversine function in the geosphere R package (Hijmans, 2024). For each unique receiver and tag combination, the temporal overlap between tag transmission and receiver deployment periods was identified. An hourly time series was generated for each overlapping period, ensuring comprehensive inclusion of both detection events and non-detection periods. The hourly detection efficiency for each receiver pair was estimated using the calculations:

Expected detections per hour = 3600 seconds / transmission interval

Efficiency = number of detections / expected detections per hour

The V9 tags transmit every 350 to 370 seconds. To account for this uncertainty in interval, hourly detection efficiency was estimated for 350 (min), 360 (mean) and 370 (max) second intervals and used in subsequent aggregation and probability estimates. Only the mean hourly detection efficiency was used within statistical models. Detection data were aggregated by unique receiver-tag-distance combinations, summing successful detections and total expected detections to provide binomial format for proportion calculations in subsequent models. To compare models and detection range predictions a binomial logistic, polynomial logistic (quadratic) and natural cubic spline (n = 2) logistic regression model was fitted to the aggregated data. The operational detection ranges were calculated by identifying the maximum distance where predicted detection probability exceeded the 50% and 10% thresholds with uncertainty estimates derived via bootstrap resampling. Akaike Information Criteria (AIC) was used to assess best model fit and 5-fold cross-validation (CV loss) for predictive performance. Model prediction accuracy was assessed using root mean square error (RMSE) and mean absolute error (MAE) between predicted detection probabilities and observed detection proportions.

***Results***

Of the four V9 Range test tags, one exhibited potential transmission issues over the course of the test period resulting in unexpected low efficiency at the 99m distance and cessation of detections in late March 2025, ~2 months before the 3 other tags (**Figure S1**). This tag also provided detection data for the furthest sample distances (1060m and 1111m) but the potential issue is unlikely to have had significant effect on these results due to the very low efficiency at >750m (**Figures S1 & S2**). The detection data for all tags were included in the analysis to maintain the sampling structure and power in the analysis, but accepting the tag issue may affect model selection and accuracy of estimations.

The detection data exhibited a high degree of separation, with high detection (≥80%) at distances ≤400m and near-zero detection (≤1%) beyond 750m. This pattern, while ecologically valid for establishing maximum detection range, precluded reliable estimation of dispersion parameters. The polynomial model demonstrated the best fit to the sharply declining detection function with the lowest AIC value (AIC = 9035; **Table S1**), outperforming both the spline (ΔAIC = 5427; **Table S1**) and binomial (ΔAIC = 52522; **Table S1**) models. Cross-validation, RMSE and MSE results supported this finding with the polynomial model showing the lowest errors (**Table S1**). The separation between high- and low-efficiency zones resulted in computational instability for quasi-likelihood measures but did not affect the models’ ability to characterize the 50% and 10% efficiency detection ranges (**Table S1; Figure S2**). The 50% detection range was estimated to be 560m (polynomial), 590m (spline) and 500m (binomial), while the 10% detection was 670m (polynomial) and 690m (spline and binomial).

**Supplementary Table S1** Summary of range test model fit comparison, range estimates and cross-validation results.

| Model | AIC | Δ AIC | 50% Detection Range (m)  (Bootstrap 95% CIs) | 10% Detection Range (m)  (Bootstrap 95% CIs) | RMSE | MAE | CV loss |
| --- | --- | --- | --- | --- | --- | --- | --- |
| Polynomial | 9035 | 0 | 560m (320-670m) | 670m (380-1500m) | 0.0066 | 0.0041 | 1938 |
| Spline (df=2) | 14462 | 5427 | 590m (240-720m) | 690m (400-840m) | 0.0110 | 0.0072 | 2060 |
| Binomial | 61557 | 52522 | 500m (290-560m) | 690m (530-800m) | 0.0609 | 0.0360 | 4389 |

Δ AIC: Difference in AIC from best-performing standard binomial model

RMSE: Root mean square error, MAE: Mean absolute error, CV Loss: Cross-validated prediction error

**Supplementary Figure S1** Hourly detection efficiency timeseries panel plot for 4 V9 tags at 12 distances from 3 receivers in the Beatrice wind farm B2 cluster.

**Supplementary Figure S2** Mean hourly detection efficient (raw data) for V9 tags with distance from acoustic receivers. Error bars represent the minimum and maximum transmission interval of the tags. Binomial (blue solid), polynomial (red dashed) and natural spline (green dash-dot) logistic model predictions with coloured dots representing the location of 50% and 10% detection probability for each.

**Supplementary Figure S3** Timeseries abacus panel plot of acoustic detections from 15 Atlantic cod and 89 haddock tagged in 2022 and 2024. Dot colour represents the receivers cluster, and the red box indicates cod identified as being predated

**Supplementary Figure S4** Timeseries abacus panel plot of acoustic detections from 15 Atlantic cod tagged in 2022 and 2024. Dot colour represents the receivers cluster, and the red box indicates cod identified as being predated

**Supplementary Figure S5 a, c & d**. Predated Atlantic cod tagged in 2022. Depth and temperature time series panel plots present Atlantic cod (dark blue) and marine mammal (light blue) detections as small circles. A change in detecting receiver (movement) is shown as larger circles with associated cluster colour (see legend). **b, d & f**. Spatial illustration of the detection location and number for each Atlantic cod across the receiver array. Small light grey filled circles = wind turbines, coloured filled circles = array cluster (see legend), green cross = release location dark blue line = fish movement & detections, light blue line = mammal movement & detections. Red cross = first detection after consumption by marine mammal and the red dotted line links the last and first detections either side of the gap in detections.

**Supplementary Figure S6 a, c & d**. Predated Atlantic cod tagged in 2024. Depth and temperature time series panel plots present Atlantic cod (dark blue) and marine mammal (light blue) detections as small circles. A change in detecting receiver (movement) is shown as larger circles with associated cluster colour (see legend). **b, d & f**. Spatial illustration of the detection location and number for each Atlantic cod across the receiver array. Small light grey filled circles = wind turbines, coloured filled circles = array cluster (see legend), green cross = release location dark blue line = fish movement & detections, light blue line = mammal movement & detections. Red cross = first detection after consumption by marine mammal and the red dotted line links the last and first detections either side of the gap in detections.

**Supplementary Figure S7 a, c & d**. Predation events of Atlantic cod tagged in 2022. Depth and temperature time series panel plots present detections after the >10 degrees increase in temperature as filled circles (dark grey shading = night, light grey = dawn/dusk). A change in cluster (large movement) is shown with associated colour (see legend). **b, d & f**. Spatial illustration of the detection location and number for each marine mammal predator across the receiver array. Small light grey filled circles = wind turbines, coloured filled circles = array cluster (see legend), green cross = cod release location, red cross = first detection after consumption by marine mammal, light blue line = mammal movement & detections.

**Supplementary Figure S8 a, c & d**. Predation events of Atlantic cod tagged in 2024. Depth and temperature time series panel plots present detections after the >10 degrees increase in temperature as filled circles (dark grey shading = night, light grey = dawn/dusk). A change in cluster (large movement) is shown with associated colour (see legend). **b, d & f**. Spatial illustration of the detection location and number for each marine mammal predator across the receiver array. Small light grey filled circles = wind turbines, coloured filled circles = array cluster (see legend), green cross = cod release location, red cross = first detection after consumption by marine mammal, light blue line = mammal movement & detections.

**Supplementary Figure S9** Box and whisker panel plots for depth and temperature detected before and after identified predation events for 6 tagged Atlantic cod.

**Supplementary Table S2** Biometrics, sensor and detection information of Atlantic cod and haddock tagged in the Moray Firth wind farms and detected for 14 or more days

| **Animal ID** | **Species** | **Predation** | **Length (cm)** | **Release year** | **First detection** | **Last detection** | **Days (dates) detected** | **Days (24hrs) at large** | **Depth (min)** | **Depth (max)** | **Depth (range)** | **Depth (var)** | **Temp (min)** | **Temp (max)** | **Temp (range)** | **Temp (var)** |
| --- | --- | --- | --- | --- | --- | --- | --- | --- | --- | --- | --- | --- | --- | --- | --- | --- |
| 65594 | Gadus morhua | Predated | 30 | 2022 | 2022-04-22 00:01:30 | 2022-11-12 04:21:04 | 31 | 204 | -0.9 | -53 | 52.4 | 8.78 | 7.5 | 37.0 | 29.50 | 6.03 |
| 65596 | Gadus morhua | Predated | 28 | 2022 | 2022-04-22 00:02:45 | 2022-09-09 22:43:38 | 141 | 141 | -0.6 | -47 | 46.3 | 2.17 | 7.5 | 37.3 | 29.81 | 4.93 |
| 65589 | Gadus morhua | Predated | 32 | 2022 | 2022-04-21 00:10:43 | 2023-01-11 10:08:15 | 246 | 265 | -0.6 | -53 | 52.7 | 0.92 | 7.7 | 37.5 | 29.81 | 2.63 |
| 70942 | Gadus morhua | Predated | 30 | 2024 | 2024-04-27 00:01:49 | 2024-07-27 19:29:57 | 72 | 92 | -0.3 | -44 | 43.6 | 1.00 | 7.8 | 35.5 | 27.61 | 0.93 |
| 70861 | Gadus morhua | Predated | 29 | 2024 | 2024-04-25 23:54:57 | 2024-08-15 17:04:32 | 48 | 112 | -5.4 | -51 | 46.0 | 11.13 | 8.0 | 37.0 | 29.03 | 7.04 |
| 70865 | Gadus morhua | Predated | 36 | 2024 | 2024-05-15 09:00:48 | 2024-08-14 19:01:31 | 61 | 91 | 0.6 | -51 | 51.8 | 11.53 | 8.6 | 35.5 | 26.83 | 2.71 |
| 79103 | Gadus morhua | Not predated | 28 | 2022 | 2022-04-22 00:01:01 | 2023-06-06 14:41:41 | 237 | 411 | -19.0 | -46 | 27.1 | 2.07 | 6.6 | 14.1 | 7.53 | 1.92 |
| 65586 | Gadus morhua | Not predated | 34 | 2022 | 2022-04-21 00:15:45 | 2023-03-25 23:06:25 | 293 | 339 | -39.1 | -52 | 13.2 | 1.12 | 6.9 | 13.8 | 6.90 | 3.49 |
| 65580 | Gadus morhua | Not predated | 36 | 2022 | 2022-04-21 00:16:03 | 2022-06-06 13:52:07 | 14 | 47 | -4.8 | -51 | 46.6 | 97.76 | 7.5 | 11.3 | 3.77 | 0.78 |
| 65606 | Gadus morhua | Not predated | 31 | 2022 | 2022-06-24 00:01:58 | 2023-06-18 14:26:12 | 301 | 360 | -49.4 | -53 | 3.9 | 0.65 | 6.8 | 13.6 | 6.90 | 4.49 |
| 70945 | Gadus morhua | Not predated | 24 | 2024 | 2024-04-27 00:02:55 | 2024-08-09 22:30:12 | 95 | 105 | -31.6 | -45 | 12.9 | 0.73 | 7.8 | 12.1 | 4.24 | 1.77 |
| 70948 | Gadus morhua | Not predated | 24 | 2024 | 2024-04-27 00:01:27 | 2024-07-04 02:16:19 | 60 | 68 | 0.0 | -76 | 75.5 | 2.61 | 7.8 | 11.0 | 3.14 | 0.56 |
| 70854 | Gadus morhua | Not predated | 32 | 2024 | 2024-04-25 13:26:35 | 2024-11-14 14:39:52 | 70 | 203 | -49.4 | -53 | 3.6 | 0.61 | 7.8 | 13.0 | 5.18 | 2.77 |
| 70864 | Gadus morhua | Not predated | 24 | 2024 | 2024-04-25 19:33:30 | 2025-03-22 00:21:09 | 68 | 330 | -45.4 | -53 | 7.2 | 3.28 | 7.5 | 13.0 | 5.49 | 2.21 |
| 70869 | Gadus morhua | Not predated | 36 | 2024 | 2024-05-04 03:55:46 | 2025-03-22 11:53:24 | 62 | 322 | -34.0 | -55 | 20.8 | 7.18 | 6.6 | 12.7 | 6.12 | 1.17 |
| 65600 | Melanogrammus aeglefinus | Not predated | 30 | 2022 | 2022-04-22 00:00:44 | 2022-11-13 12:30:18 | 162 | 206 | -43.0 | -47 | 3.6 | 0.68 | 7.4 | 14.0 | 6.59 | 2.85 |
| 65601 | Melanogrammus aeglefinus | Not predated | 30 | 2022 | 2022-04-22 00:05:29 | 2022-10-25 23:53:50 | 126 | 187 | -42.1 | -46 | 3.9 | 0.63 | 7.4 | 13.6 | 6.28 | 3.76 |
| 65602 | Melanogrammus aeglefinus | Not predated | 30 | 2022 | 2022-04-22 00:41:39 | 2022-11-12 10:36:41 | 154 | 204 | -42.4 | -46 | 3.6 | 0.63 | 7.5 | 14.1 | 6.59 | 2.82 |
| 65603 | Melanogrammus aeglefinus | Not predated | 30 | 2022 | 2022-04-22 00:17:33 | 2022-11-13 14:28:31 | 157 | 206 | -42.4 | -46 | 3.9 | 0.71 | 7.5 | 14.3 | 6.75 | 3.31 |
| 65590 | Melanogrammus aeglefinus | Not predated | 31 | 2022 | 2022-04-22 00:14:03 | 2023-03-20 04:37:37 | 151 | 332 | -42.7 | -46 | 3.6 | 0.67 | 7.7 | 14.3 | 6.59 | 3.53 |
| 65592 | Melanogrammus aeglefinus | Not predated | 30 | 2022 | 2022-04-22 00:17:28 | 2022-08-26 00:51:59 | 102 | 126 | -42.7 | -46 | 3.6 | 0.66 | 7.7 | 13.0 | 5.33 | 1.97 |
| 65573 | Melanogrammus aeglefinus | Not predated | 26 | 2022 | 2022-04-20 00:06:27 | 2022-08-09 14:28:22 | 108 | 112 | -49.4 | -53 | 3.3 | 0.59 | 7.4 | 12.2 | 4.86 | 1.80 |
| 65574 | Melanogrammus aeglefinus | Not predated | 34 | 2022 | 2022-04-20 00:14:32 | 2023-06-15 10:05:51 | 146 | 421 | -48.8 | -52 | 3.6 | 0.61 | 6.9 | 13.0 | 6.12 | 2.27 |
| 65575 | Melanogrammus aeglefinus | Not predated | 30 | 2022 | 2022-04-20 00:01:37 | 2022-08-28 21:32:14 | 116 | 131 | -49.0 | -53 | 3.9 | 0.59 | 7.4 | 13.0 | 5.65 | 2.45 |
| 65576 | Melanogrammus aeglefinus | Not predated | 29 | 2022 | 2022-04-20 00:13:52 | 2022-11-16 01:44:06 | 191 | 210 | -49.0 | -53 | 3.6 | 0.64 | 7.4 | 13.6 | 6.28 | 3.52 |
| 65578 | Melanogrammus aeglefinus | Not predated | 25 | 2022 | 2022-04-20 00:03:19 | 2022-11-12 12:14:26 | 181 | 207 | -49.0 | -53 | 3.6 | 0.62 | 7.4 | 13.8 | 6.43 | 3.82 |
| 65568 | Melanogrammus aeglefinus | Not predated | 28 | 2022 | 2022-04-20 00:04:38 | 2022-11-16 10:02:53 | 23 | 210 | -49.4 | -53 | 3.3 | 0.54 | 7.4 | 13.3 | 5.96 | 0.42 |
| 65569 | Melanogrammus aeglefinus | Not predated | 28 | 2022 | 2022-04-20 00:14:12 | 2023-06-15 10:59:01 | 121 | 421 | -49.0 | -53 | 3.6 | 0.57 | 6.8 | 13.3 | 6.59 | 1.89 |
| 65571 | Melanogrammus aeglefinus | Not predated | 28 | 2022 | 2022-04-20 00:13:43 | 2023-06-14 07:17:17 | 165 | 420 | -48.8 | -53 | 3.9 | 0.68 | 6.9 | 13.8 | 6.90 | 5.01 |
| 65572 | Melanogrammus aeglefinus | Not predated | 37 | 2022 | 2022-04-20 00:05:35 | 2022-11-16 00:16:43 | 70 | 210 | -49.0 | -53 | 3.6 | 0.64 | 7.4 | 13.6 | 6.28 | 1.60 |
| 65562 | Melanogrammus aeglefinus | Not predated | 26 | 2022 | 2022-04-20 00:01:15 | 2023-04-17 00:35:50 | 196 | 362 | -49.0 | -53 | 3.9 | 0.59 | 6.9 | 13.6 | 6.75 | 3.42 |
| 65564 | Melanogrammus aeglefinus | Not predated | 32 | 2022 | 2022-04-20 00:16:23 | 2022-11-16 01:43:19 | 175 | 210 | -49.0 | -53 | 3.6 | 0.62 | 7.4 | 13.6 | 6.28 | 3.08 |
| 65565 | Melanogrammus aeglefinus | Not predated | 30 | 2022 | 2022-04-20 00:05:47 | 2023-06-16 11:47:50 | 259 | 422 | -49.0 | -53 | 3.6 | 0.56 | 6.8 | 13.5 | 6.75 | 3.70 |
| 65566 | Melanogrammus aeglefinus | Not predated | 26 | 2022 | 2022-04-20 00:00:52 | 2022-10-29 10:00:18 | 139 | 192 | -49.0 | -53 | 3.6 | 0.68 | 7.4 | 13.8 | 6.43 | 4.81 |
| 65567 | Melanogrammus aeglefinus | Not predated | 38 | 2022 | 2022-04-20 00:27:41 | 2023-06-16 10:36:51 | 392 | 422 | -49.0 | -53 | 3.9 | 0.63 | 6.8 | 13.6 | 6.90 | 4.41 |
| 65559 | Melanogrammus aeglefinus | Not predated | 28 | 2022 | 2022-04-20 00:35:11 | 2023-03-06 02:34:28 | 205 | 320 | -49.0 | -53 | 3.6 | 0.65 | 7.4 | 13.8 | 6.43 | 2.96 |
| 65560 | Melanogrammus aeglefinus | Not predated | 28 | 2022 | 2022-04-20 00:13:02 | 2022-05-15 08:16:14 | 23 | 25 | -49.4 | -52 | 3.0 | 0.61 | 7.4 | 8.3 | 0.94 | 0.10 |
| 65561 | Melanogrammus aeglefinus | Not predated | 26 | 2022 | 2022-04-20 00:06:17 | 2023-05-26 00:58:56 | 214 | 401 | -49.0 | -53 | 3.6 | 0.62 | 6.9 | 13.6 | 6.75 | 3.27 |
| 65614 | Melanogrammus aeglefinus | Not predated | 35 | 2022 | 2022-06-27 03:45:15 | 2022-09-07 20:37:02 | 67 | 73 | -29.2 | -50 | 20.8 | 1.55 | 10.5 | 13.6 | 3.14 | 0.57 |
| 65579 | Melanogrammus aeglefinus | Not predated | 32 | 2022 | 2022-04-21 01:05:40 | 2023-06-16 07:02:49 | 220 | 421 | -46.9 | -57 | 9.9 | 1.03 | 7.7 | 13.6 | 5.96 | 3.17 |
| 65607 | Melanogrammus aeglefinus | Not predated | 38 | 2022 | 2022-06-24 00:01:13 | 2023-05-02 13:29:41 | 108 | 313 | -49.4 | -53 | 3.6 | 0.60 | 7.1 | 13.6 | 6.59 | 1.54 |
| 65608 | Melanogrammus aeglefinus | Not predated | 30 | 2022 | 2022-06-24 00:09:06 | 2022-11-12 05:39:01 | 122 | 141 | -49.4 | -53 | 3.6 | 0.67 | 10.4 | 13.6 | 3.29 | 1.02 |
| 65612 | Melanogrammus aeglefinus | Not predated | 34 | 2022 | 2022-06-24 00:13:48 | 2022-09-24 22:14:29 | 80 | 93 | -48.8 | -53 | 3.9 | 0.73 | 10.4 | 13.6 | 3.29 | 0.61 |
| 70916 | Melanogrammus aeglefinus | Not predated | 26 | 2024 | 2024-05-27 04:44:47 | 2024-11-19 12:05:23 | 21 | 176 | -15.9 | -60 | 44.5 | 29.53 | 8.9 | 10.4 | 1.41 | 0.14 |
| 70917 | Melanogrammus aeglefinus | Not predated | 32 | 2024 | 2024-04-28 20:24:33 | 2024-08-26 16:39:10 | 121 | 120 | -3.3 | -48 | 44.8 | 68.50 | 7.8 | 12.7 | 4.86 | 2.05 |
| 70918 | Melanogrammus aeglefinus | Not predated | 32 | 2024 | 2024-04-27 01:43:06 | 2024-06-28 04:00:34 | 26 | 62 | -3.9 | -53 | 48.8 | 72.46 | 7.8 | 10.5 | 2.67 | 0.31 |
| 70919 | Melanogrammus aeglefinus | Not predated | 32 | 2024 | 2024-04-28 14:49:44 | 2025-05-16 18:09:48 | 123 | 383 | -9.3 | -56 | 46.6 | 136.88 | 6.6 | 12.2 | 5.65 | 0.88 |
| 70920 | Melanogrammus aeglefinus | Not predated | 32 | 2024 | 2024-04-27 00:57:07 | 2025-05-15 07:31:22 | 254 | 383 | -26.2 | -44 | 18.1 | 0.72 | 6.8 | 13.0 | 6.28 | 4.10 |
| 70926 | Melanogrammus aeglefinus | Not predated | 32 | 2024 | 2024-04-28 18:05:48 | 2025-01-24 06:53:21 | 30 | 271 | -10.8 | -55 | 43.9 | 106.15 | 7.5 | 11.3 | 3.77 | 1.59 |
| 70927 | Melanogrammus aeglefinus | Not predated | 31 | 2024 | 2024-04-27 06:07:28 | 2024-08-03 16:02:16 | 71 | 98 | -0.9 | -50 | 49.4 | 62.74 | 8.0 | 12.2 | 4.24 | 0.71 |
| 70928 | Melanogrammus aeglefinus | Not predated | 30 | 2024 | 2024-06-14 05:37:13 | 2024-07-19 17:26:54 | 19 | 35 | -12.0 | -53 | 40.6 | 136.11 | 10.0 | 11.4 | 1.41 | 0.11 |
| 70879 | Melanogrammus aeglefinus | Not predated | 32 | 2024 | 2024-04-24 02:46:20 | 2025-05-17 00:15:17 | 198 | 388 | -1.8 | -53 | 51.5 | 97.56 | 6.6 | 13.0 | 6.43 | 1.80 |
| 70888 | Melanogrammus aeglefinus | Not predated | 28 | 2024 | 2024-05-13 22:30:55 | 2025-05-14 11:22:50 | 366 | 366 | -45.7 | -54 | 7.8 | 0.62 | 6.8 | 13.0 | 6.28 | 3.57 |
| 70889 | Melanogrammus aeglefinus | Not predated | 31 | 2024 | 2024-04-26 00:55:56 | 2024-11-12 06:19:03 | 49 | 200 | -49.0 | -52 | 3.3 | 0.62 | 7.8 | 12.4 | 4.55 | 0.91 |
| 70890 | Melanogrammus aeglefinus | Not predated | 37 | 2024 | 2024-04-26 04:48:13 | 2024-07-04 10:26:25 | 67 | 69 | -9.0 | -54 | 45.4 | 184.44 | 7.8 | 11.3 | 3.45 | 0.81 |
| 70891 | Melanogrammus aeglefinus | Not predated | 30 | 2024 | 2024-04-26 06:31:29 | 2024-08-20 03:27:31 | 94 | 116 | -9.3 | -56 | 46.6 | 114.35 | 7.8 | 12.7 | 4.86 | 1.51 |
| 70895 | Melanogrammus aeglefinus | Not predated | 34 | 2024 | 2024-05-24 03:40:48 | 2025-05-17 07:18:48 | 259 | 358 | -15.3 | -55 | 39.4 | 23.59 | 7.4 | 13.0 | 5.65 | 2.82 |
| 70896 | Melanogrammus aeglefinus | Not predated | 28 | 2024 | 2024-04-26 00:01:06 | 2024-05-19 22:59:36 | 11 | 24 | -34.0 | -54 | 20.5 | 3.36 | 7.8 | 8.8 | 0.94 | 0.08 |
| 70897 | Melanogrammus aeglefinus | Not predated | 29 | 2024 | 2024-04-26 01:25:35 | 2025-02-14 00:19:13 | 293 | 294 | -5.1 | -55 | 50.0 | 59.66 | 7.1 | 13.0 | 5.96 | 3.07 |
| 70898 | Melanogrammus aeglefinus | Not predated | 30 | 2024 | 2024-04-26 00:03:37 | 2024-10-20 03:21:50 | 164 | 177 | -8.7 | -56 | 46.9 | 78.51 | 7.8 | 13.0 | 5.18 | 1.48 |
| 70899 | Melanogrammus aeglefinus | Not predated | 30 | 2024 | 2024-04-26 00:00:42 | 2025-05-15 11:04:23 | 346 | 384 | -12.6 | -55 | 42.4 | 5.89 | 6.8 | 13.0 | 6.28 | 3.91 |
| 70900 | Melanogrammus aeglefinus | Not predated | 28 | 2024 | 2024-04-26 00:06:05 | 2024-08-01 20:26:38 | 94 | 98 | -12.3 | -56 | 43.3 | 69.27 | 7.8 | 11.6 | 3.77 | 1.12 |
| 70901 | Melanogrammus aeglefinus | Not predated | 30 | 2024 | 2024-04-26 04:02:25 | 2024-08-18 22:44:35 | 73 | 115 | -13.5 | -55 | 41.2 | 104.79 | 7.8 | 11.9 | 4.08 | 1.11 |
| 70902 | Melanogrammus aeglefinus | Not predated | 33 | 2024 | 2024-04-26 04:23:38 | 2025-02-21 02:43:10 | 278 | 301 | -13.2 | -55 | 41.8 | 26.92 | 6.6 | 13.0 | 6.43 | 2.42 |
| 70903 | Melanogrammus aeglefinus | Not predated | 32 | 2024 | 2024-04-26 00:02:24 | 2024-09-08 05:02:03 | 135 | 135 | -2.4 | -54 | 51.8 | 71.46 | 7.8 | 13.0 | 5.18 | 1.77 |
| 70905 | Melanogrammus aeglefinus | Not predated | 26 | 2024 | 2024-05-13 22:17:22 | 2025-05-14 11:22:43 | 365 | 366 | -46.3 | -54 | 7.5 | 0.60 | 6.8 | 13.0 | 6.28 | 3.24 |
| 70906 | Melanogrammus aeglefinus | Not predated | 32 | 2024 | 2024-04-26 03:45:14 | 2024-11-15 09:58:23 | 111 | 203 | -9.6 | -53 | 43.6 | 58.76 | 7.8 | 13.0 | 5.18 | 1.70 |
| 70907 | Melanogrammus aeglefinus | Not predated | 32 | 2024 | 2024-04-26 11:21:08 | 2025-05-17 12:15:42 | 217 | 386 | 0.0 | -56 | 56.3 | 193.71 | 6.6 | 13.0 | 6.43 | 4.97 |
| 70908 | Melanogrammus aeglefinus | Not predated | 30 | 2024 | 2024-04-26 00:02:56 | 2025-05-14 04:24:58 | 125 | 383 | -49.0 | -53 | 3.6 | 0.63 | 7.4 | 12.9 | 5.49 | 2.71 |
| 70910 | Melanogrammus aeglefinus | Not predated | 29 | 2024 | 2024-04-26 01:00:43 | 2024-06-27 06:11:40 | 35 | 62 | -8.1 | -55 | 46.6 | 88.33 | 7.8 | 10.5 | 2.67 | 0.26 |
| 70912 | Melanogrammus aeglefinus | Not predated | 30 | 2024 | 2024-04-26 03:54:10 | 2025-02-22 18:30:00 | 242 | 303 | 0.9 | -55 | 55.7 | 135.24 | 6.9 | 13.0 | 6.12 | 2.45 |
| 70996 | Melanogrammus aeglefinus | Not predated | 31 | 2024 | 2024-04-29 00:00:23 | 2025-02-09 04:52:49 | 239 | 286 | -12.0 | -51 | 38.8 | 56.46 | 7.4 | 13.0 | 5.65 | 2.17 |
| 71001 | Melanogrammus aeglefinus | Not predated | 30 | 2024 | 2024-04-29 03:37:16 | 2024-09-07 16:31:26 | 66 | 132 | -21.4 | -48 | 27.1 | 5.99 | 8.0 | 13.0 | 5.02 | 1.93 |
| 71002 | Melanogrammus aeglefinus | Not predated | 32 | 2024 | 2024-04-30 05:32:31 | 2024-09-07 02:13:53 | 131 | 130 | 0.0 | -50 | 50.0 | 71.56 | 8.0 | 13.2 | 5.18 | 2.09 |
| 71004 | Melanogrammus aeglefinus | Not predated | 36 | 2024 | 2024-05-01 09:59:25 | 2025-01-05 02:02:36 | 242 | 249 | -15.9 | -50 | 34.3 | 18.08 | 8.0 | 13.2 | 5.18 | 1.97 |
| 71008 | Melanogrammus aeglefinus | Not predated | 30 | 2024 | 2024-04-29 01:48:01 | 2025-05-15 08:01:23 | 297 | 381 | -16.6 | -47 | 30.1 | 0.90 | 6.9 | 13.0 | 6.12 | 1.62 |
| 71012 | Melanogrammus aeglefinus | Not predated | 32 | 2024 | 2024-04-29 06:35:16 | 2025-05-14 07:47:49 | 209 | 380 | -36.1 | -50 | 14.1 | 0.62 | 6.6 | 13.0 | 6.43 | 1.63 |
| 71015 | Melanogrammus aeglefinus | Not predated | 32 | 2024 | 2024-04-29 21:09:19 | 2025-01-24 14:47:09 | 96 | 270 | -5.4 | -51 | 45.7 | 118.64 | 7.4 | 12.9 | 5.49 | 1.84 |
| 71018 | Melanogrammus aeglefinus | Not predated | 32 | 2024 | 2024-04-29 09:43:03 | 2024-11-17 07:52:59 | 66 | 202 | -11.7 | -54 | 42.4 | 65.25 | 8.0 | 12.7 | 4.71 | 1.16 |
| 71019 | Melanogrammus aeglefinus | Not predated | 30 | 2024 | 2024-04-29 19:29:47 | 2024-09-30 17:14:31 | 91 | 154 | -1.8 | -54 | 52.1 | 98.79 | 8.0 | 13.0 | 5.02 | 1.19 |
| 71022 | Melanogrammus aeglefinus | Not predated | 31 | 2024 | 2024-04-29 03:17:42 | 2025-04-29 17:29:05 | 205 | 366 | -41.5 | -55 | 13.8 | 0.58 | 7.8 | 13.0 | 5.18 | 2.12 |
| 71024 | Melanogrammus aeglefinus | Not predated | 29 | 2024 | 2024-04-29 02:15:46 | 2025-05-10 19:20:44 | 173 | 377 | -44.2 | -48 | 3.9 | 0.54 | 6.9 | 13.0 | 6.12 | 2.02 |
| 71029 | Melanogrammus aeglefinus | Not predated | 30 | 2024 | 2024-04-29 22:02:47 | 2025-01-27 21:25:16 | 254 | 273 | -5.1 | -54 | 48.4 | 25.53 | 7.2 | 13.0 | 5.81 | 2.57 |
| 71030 | Melanogrammus aeglefinus | Not predated | 28 | 2024 | 2024-04-29 00:15:56 | 2024-10-14 23:10:15 | 49 | 169 | -3.6 | -54 | 50.5 | 122.17 | 7.8 | 12.6 | 4.71 | 1.29 |
| 70847 | Melanogrammus aeglefinus | Not predated | 32 | 2024 | 2024-04-23 00:00:20 | 2025-03-09 06:02:22 | 197 | 320 | -47.8 | -55 | 7.5 | 0.75 | 7.8 | 13.0 | 5.18 | 2.45 |
| 70849 | Melanogrammus aeglefinus | Not predated | 30 | 2024 | 2024-04-23 00:01:25 | 2024-09-19 10:14:45 | 128 | 149 | -48.1 | -56 | 8.1 | 0.69 | 7.8 | 13.0 | 5.18 | 2.09 |
| 70857 | Melanogrammus aeglefinus | Not predated | 30 | 2024 | 2024-04-23 15:22:36 | 2025-05-14 08:15:48 | 362 | 386 | -25.3 | -57 | 31.3 | 0.75 | 6.6 | 13.0 | 6.43 | 2.88 |
| 70858 | Melanogrammus aeglefinus | Not predated | 26 | 2024 | 2024-04-23 00:46:04 | 2025-05-15 21:03:55 | 277 | 388 | -4.2 | -54 | 49.6 | 101.45 | 6.4 | 13.0 | 6.59 | 1.99 |
| 70863 | Melanogrammus aeglefinus | Not predated | 34 | 2024 | 2024-04-24 11:27:39 | 2024-08-23 10:51:51 | 18 | 121 | -8.4 | -57 | 48.8 | 76.02 | 7.8 | 12.7 | 4.86 | 0.57 |
| 70866 | Melanogrammus aeglefinus | Not predated | 30 | 2024 | 2024-04-23 00:44:08 | 2024-10-31 12:20:05 | 95 | 191 | -3.0 | -54 | 51.1 | 100.57 | 7.8 | 12.9 | 5.02 | 1.10 |
| 70958 | Melanogrammus aeglefinus | Not predated | 31 | 2024 | 2024-06-07 02:59:00 | 2025-05-29 18:14:51 | 256 | 357 | -18.7 | -58 | 39.7 | 18.05 | 7.5 | 13.0 | 5.49 | 2.48 |
| 70961 | Melanogrammus aeglefinus | Not predated | 34 | 2024 | 2024-05-21 01:30:08 | 2024-12-01 20:52:06 | 24 | 195 | -17.8 | -55 | 37.3 | 38.75 | 8.6 | 10.2 | 1.57 | 0.20 |
| 70963 | Melanogrammus aeglefinus | Not predated | 36 | 2024 | 2024-04-28 00:09:35 | 2025-04-28 07:50:41 | 331 | 365 | -7.5 | -57 | 49.4 | 11.04 | 6.4 | 13.0 | 6.59 | 4.06 |
| 70969 | Melanogrammus aeglefinus | Not predated | 34 | 2024 | 2024-04-28 00:02:07 | 2025-02-12 20:12:18 | 22 | 291 | -2.4 | -55 | 52.4 | 142.20 | 6.9 | 9.6 | 2.67 | 0.69 |
| 70972 | Melanogrammus aeglefinus | Not predated | 30 | 2024 | 2024-04-28 14:14:13 | 2025-03-26 19:05:49 | 69 | 332 | -8.7 | -57 | 47.8 | 137.84 | 7.2 | 12.9 | 5.65 | 1.36 |
| 70975 | Melanogrammus aeglefinus | Not predated | 37 | 2024 | 2024-04-30 05:05:39 | 2025-05-17 23:05:27 | 86 | 383 | -21.7 | -58 | 36.7 | 80.52 | 6.8 | 12.6 | 5.81 | 1.89 |
| 70976 | Melanogrammus aeglefinus | Not predated | 38 | 2024 | 2024-04-28 01:28:15 | 2024-10-06 04:26:42 | 48 | 161 | -41.8 | -45 | 3.3 | 0.52 | 8.0 | 13.0 | 5.02 | 1.35 |
| 70977 | Melanogrammus aeglefinus | Not predated | 24 | 2024 | 2024-04-28 00:59:07 | 2025-04-07 19:30:11 | 106 | 345 | -49.0 | -57 | 8.4 | 0.74 | 8.0 | 13.0 | 5.02 | 1.17 |
| 70987 | Melanogrammus aeglefinus | Not predated | 32 | 2024 | 2024-04-28 03:26:30 | 2024-05-26 22:42:29 | 22 | 29 | -46.9 | -54 | 7.5 | 2.62 | 8.0 | 8.9 | 0.94 | 0.04 |
| 70988 | Melanogrammus aeglefinus | Not predated | 32 | 2024 | 2024-04-28 07:31:35 | 2024-11-28 03:00:00 | 128 | 214 | -8.1 | -53 | 44.5 | 65.76 | 7.8 | 26.8 | 18.98 | 1.20 |
| 70989 | Melanogrammus aeglefinus | Not predated | 32 | 2024 | 2024-04-28 01:02:42 | 2024-06-10 10:43:25 | 14 | 43 | -11.7 | -53 | 41.5 | 75.14 | 7.8 | 10.5 | 2.67 | 0.34 |
| 70991 | Melanogrammus aeglefinus | Not predated | 32 | 2024 | 2024-04-28 00:40:03 | 2024-11-17 10:39:05 | 186 | 203 | -3.6 | -55 | 51.5 | 39.53 | 7.8 | 13.0 | 5.18 | 1.58 |
| 70992 | Melanogrammus aeglefinus | Not predated | 30 | 2024 | 2024-04-28 01:11:22 | 2024-05-26 22:48:29 | 22 | 29 | -46.6 | -54 | 7.8 | 3.00 | 8.0 | 8.9 | 0.94 | 0.04 |
| 70993 | Melanogrammus aeglefinus | Not predated | 30 | 2024 | 2024-04-28 00:05:29 | 2025-05-29 02:41:54 | 211 | 396 | -49.6 | -54 | 3.9 | 0.65 | 7.4 | 13.0 | 5.65 | 3.74 |

**Supplementary Table S3** Probability (Fisher exact test) of prey selection based on observed predation of tagged Atlantic cod and haddock

| Probability of 0 haddock predation events detection with equal species risk of 0.4 based on:  Cod tagged: 15, with 6 detected predation events (0.4); Haddock tagged: 89 with 0 detected predation events | | | |
| --- | --- | --- | --- |
| Question | Method | Formal Test | p-value |
| Given there were 6 total detected predation events, how likely is it that all 6 were on cod if there is no prey selection? | Conditional on total detected predation events (hypergeometric / Fisher logic) | Fisher exact test (one-sided) | 0.0000033 |
